# Exosomal lncRNA SNHG10 derived from colorectal cancer cells suppresses natural killer cell cytotoxicity by upregulating INHBC

**DOI:** 10.1186/s12935-021-02221-2

**Published:** 2021-10-12

**Authors:** Yiwen Huang, Yanbo Luo, Wentao Ou, Yuanyuan Wang, Dong Dong, Xiaowen Peng, Yuqi Luo

**Affiliations:** 1 Department of Emergency, Nansha Hospital, Guangzhou First People’s Hospital, School of Medicine, Southern China University of Technology, Guangzhou, Guangdong China; 2 Department of Gastrointestinal and Hepatobiliary Surgery, Shenzhen Longhua District Central Hospital, No. 187, Guanlan Road, Longhua District, Shenzhen, 518110 Guangdong Province China; 3 Department of General Surgery, Nansha Hospital, Guangzhou First People’s Hospital, School of Medicine, Southern China University of Technology, Guangzhou, Guangdong China; 4 Department of Neurology, Nansha Hospital, Guangzhou First People’s Hospital, School of Medicine, Southern China University of Technology, Guangzhou, Guangdong China; 5 Department of Laboratory Medicine, Guangzhou First People’s Hospital, School of Medicine, Nansha Hospital, Southern China University of Technology, Guangzhou, Guangdong China

**Keywords:** Colorectal cancer, Exosomal lncRNA, Epithelial–mesenchymal transition, Immune escape, Natural killer cells

## Abstract

**Background:**

Exosome-mediated crosstalk between cancer cells and immune cells contributes to tumor growth. In this study, we investigated the mechanism underlying the exosome-mediated immune escape of colorectal cancer (CRC) cells from natural killer (NK) cells via the transfer of long noncoding RNAs (lncRNAs).

**Methods:**

An epithelial–mesenchymal transition (EMT) model of SW480 cells was established by transforming growth factor beta (TGF-β), followed by the assessment of the effect of EMT-derived exosomes (EMT-exo) on the functions of NK cells. RNA sequencing was performed to identify exosomal lncRNAs and target genes. The function of exosomal lncRNAs in tumor growth was further verified in vivo.

**Results:**

EMT-exo suppressed the proliferation, cytotoxicity, IFN-γ production, and perforin-1 and granzyme B secretion of NK cells. RNA sequencing revealed that SNHG10 expression was upregulated in EMT-exo compared with that in non-EMT-exo. Moreover, SNHG10 expression was upregulated in tumor tissues in CRC, which was associated with poor prognosis. Overexpression of SNHG10 in exosomes (oe-lnc-SNHG10 exo) significantly suppressed the viability and cytotoxicity of NK cells. Transcriptome sequencing of NK cells revealed that the expression levels of 114 genes were upregulated in the oe-lnc-SNHG10 exo group, including inhibin subunit beta C (*INHBC*), which was involved in the TGF-β signaling pathway. Si-INHBC treatment abrogated the effect of oe-lnc-SNHG10 exo on NK cells. oe-lnc-SNHG10 exo induced tumor growth and upregulated *INHBC* expression in mice and downregulated the expression of perforin, granzyme B, and NK1.1 in tumor tissues.

**Conclusions:**

The CRC cell-derived exosomal lncRNA SNHG10 suppresses the function of NK cells by upregulating *INHBC* expression. This study provides evidence that exosomal lncRNAs contribute to immune escape by inducing NK cell inhibition and proposes a potential treatment strategy for CRC.

**Supplementary Information:**

The online version contains supplementary material available at 10.1186/s12935-021-02221-2.

## Introduction

Colorectal cancer (CRC) is the fourth most common human malignant tumor and the second most common cause of cancer-related mortality worldwide [1]. In 2014, the age-standardized incidence and mortality rate of CRC were 17.52 per 100,000 cases and 7.91 per 100,000 cases, respectively. In China, CRC is the fifth most commonly diagnosed cancer and one of the most common causes of cancer-related mortality [2]. A lack of sensitivity to chemotherapy and metastasis to the liver contribute to most cases of treatment failure and death among patients with CRC [3]. Importantly, as described by Tang et al., cancer immune escape is a major obstacle to tumor metastasis and immunotherapy [4]. However, it is not exactly clear how CRC cells escape immune cell monitoring to achieve metastasis and invasion. Thus, it is necessary to elucidate the molecular mechanism underlying the immune escape of CRC cells to reduce the burden on patients.

Natural killer (NK) cells are an important component of the body’s natural immune system and play a surveillance role in tumors by killing cancer cells directly or by secreting cytokines following their activation [5]. NK cells also contribute to the inhibition of tumor growth and metastasis [6]. However, an increasing number of studies have demonstrated that NK cell function is suppressed or dysfunctional in tumors, such as a significant reduction in the proportion of NK cells in esophageal squamous cell carcinoma, resulting in immune escape [7]. Chan et al. proved that exposure to keratin-14 + cancer cells leads to the loss of the cytotoxic ability of NK cells and promotes metastatic outgrowth [8]. Wang et al. found that breast cancer stem-like cells inhibited the antitumor activity of NK cells and activated platelets to promote the metastasis of cancer cells [9]. However, the functional mechanism underlying the inhibition of CRC tumors by NK cells remains unclear.

The literature suggests that long noncoding RNAs (lncRNAs), a type of RNA with a transcriptional length of > 200 nucleotides, are closely related to the apoptosis, metastasis, and immune regulation of tumor cells [10]. A previous study reported that the lncRNA Pvt1 inhibited tumor progression *in vivo* by regulating the immunosuppressive activity of granulocytic myeloid-derived suppressor cells [11]. Huang et al. demonstrated that the lncRNA NKILA enhanced the sensitivity of T cells to activation-induced cell death to promote tumor immune evasion [12]. These results indicate that lncRNAs play a non-negligible role in immune regulation. However, few studies have demonstrated that lncRNAs are also involved in the functional regulation of NK cells. For example, lnc-CD56, which is an NK-specific lncRNA, upregulated the expression of CD56 in primary NK cells [13]. Therefore, a better understanding of the role and mechanisms of lncRNAs in NK cells or tumor immunity can facilitate optimization of the tumor targeting of NK cells.

The cellular crosstalk that occurs during physiological processes such as tumorigenesis and epithelial–mesenchymal transformation (EMT) are mediated by intercellular messenger particles; among which exosomes have recently been reported to play an important role in the crosstalk between tumor cells and immune cells. For example, CRC-cell-derived exosomes were shown to trigger the secretion of greater amounts of tumor necrosis factor and monocyte chemoattractant protein 1 (MCP-1) by macrophages to promote the development of CRC [14]. In turn, the CRC-derived exosomes promoted the differentiation of monocytes to macrophages [15]. In recent years, the role of exosomes derived from metastasis-related programming of EMT cells in tumor immune regulation by transferring biological signaling molecules has been widely gained attention. Ni et al. proved that exosomes derived from breast cancer tissues transmitted lncRNA SNHG16, leading to the induction of CD73 + γδ1 Treg cells [16]. Yang et al. demonstrated that tumor-derived exosomal miRNA-106b-5p activated M2-subtype tumor-associated macrophage interaction and EMT-tumor cells, leading to the acceleration of CRC metastasis [17]. Zhang et al. found that exosomal circUHRF1 derived from hepatocellular carcinoma cells induced NK cell exhaustion and caused anti-PD-1 resistance [18]. Vulpis et al. proposed that tumor exosomes are new players in the regulation of the NK cell response [19]; however, it remains unclear whether EMT metastatic exosomes derived from tumor cells mediate NK cell function.

This study aimed to explore the function and mechanism of exosomes secreted by metastatic CRC cells in the immune inhibition of NK cells. We constructed an EMT SW480 cell model induced by TGF-β and isolated the exosomes derived from EMT SW480 cells. The effect of exosomes on NK cells was assessed based on their proliferation, cytotoxicity, IFN-γ production, and expression levels of perforin and granzyme B. The key lncRNAs carried by exosomes and their target genes were identified using RNA sequencing, and their role in NK cytotoxicity was investigated in vitro and in vivo. Finally, the prognostic value of potential target genes was explored.

## Materials and methods

### Patients

Thirty CRC tumors and 30 paired paracancerous tissues were collected from patients at the Guangzhou First People’s Hospital from June 2015 to May 2019. None of the patients received treatment before surgery, and all signed an informed consent form. This study was approved by the Ethics Committee of The Guangzhou First People’s Hospital.

### Cell culture and EMT model construction

The human CRC cell line SW480 (RRID: CVCL_0546) (Procell, CL-0223, China) and the human NK cell line NK92-MI (an interleukin [IL]-2-independent NK cell line) (RRID: CVCL_3755) (Procell, CL-0533, China) were obtained from Procell Life Science Technology. The RPMI-1640 medium (10-040-CVR, CORNING, China) containing 10% fetal bovine serum (FBS; 10099-141, GIBCO, China) and 1% penicillin–streptomycin (PS; E607011, Sangon Biotech, China) was used to culture SW480 cells. The alpha modification of minimum essential medium (MEMα; CM-0533, Procell, China), containing 12.5% FBS, 1% PS, 0.2 mM inositol, 0.02 mM folic acid, 0.1 mM β-mercaptoethanol, and horse serum (164,215, Procell, China) was used to culture NK92-MI cells. All cells were maintained in an incubator supplemented with 5% CO_2_ at 37 °C. In addition, to construct the EMT model, 10 ng/mL TGF-β was added to the medium, and the cells were collected 72 h later for subsequent assays. All experiments were performed using mycoplasma-free cells, and all human cell lines were authenticated using short tandem repeat (STR) profiling within the last 3 years.

### Isolation and identification of exosomes

The supernatant of SW480 cell cultures was collected for the isolation of exosomes via differential centrifugation, as described previously [20]. The total protein quantitation of exosomes was performed using the BCA assay kit (Thermo Scientific, USA), and the concentration of each sample was adjusted to 200 µg/mL. Next, the purified exosomes were immediately fixed with 4% glutaraldehyde and 1% osmium tetroxide, and a drop of the suspension was placed on Formvar/carbon-coated electron microscopy grids and allowed to stand for 5 min. The exosomes were then stained with 10% uranium acetate for 5 min and imaged using a transmission electron microscope (TEM). Subsequently, the distribution, size, and number of particles were determined via nanoparticle-tracking analysis (NTA) using the ZetaView Nanoparticle Tracking instrument (Particle Metrix, Germany). Briefly, approximately 0.5 mL of the exosome sample diluted with phosphate-buffered solution (PBS) (1:1000) was introduced into the ZetaView Nanoparticle Tracking system, and three cycles were performed by scanning 11 cell positions and capturing 60 frames per position under conditions of 25 °C cell temperature, pH 7.0, 15000.00 µS/cm sensor conductivity, and embedded laser at 488 nm. The videos were analyzed using ZetaView software.

### qRT-PCR analysis

TRIzol reagent (Invitrogen Life Technologies, Inc., USA) was used to isolate total RNA from SW480 and NK92-MI cells according to the manufacturer’s instructions. A microspectrophotometer (Tiangen Biotech Co., Ltd., China) was used to determine the concentration and purity of RNA, and qualified RNA was frozen at – 80 °C for subsequent experiments. The RNA was reverse transcribed into first-strand cDNA using the RevertAid First Strand cDNA Synthesis Kit (Thermo Fisher Scientific, Inc.). Subsequently, qRT-PCR was performed using the FastStart Universal SYBR Green Master Mix on a QuantStudio 6 Flex Real-Time PCR System (Thermo Fisher Scientific, Inc.) according to the manufacturer’s instructions. PCR cycling was conducted using the following conditions: 95 °C for 10 min; followed by 45 cycles of 95 °C for 15 s and 60 °C for 60 s; dissociation at 95 °C for 10 s; 60 °C for 60 s; 95 °C for 15 s. All primers used are listed in Additional file 3: Tables S1 and were synthesized by Sangon Biotech (Shanghai, China). *GAPDH* was used to normalize gene expression, which was measured using the 2^−ΔΔCq^ method.

### Western blotting

Total proteins were extracted using RIPA lysis buffer (Thermo Fisher) and analyzed using a bicinchoninic acid Protein Assay Kit (Thermo Scientific, USA). An equal amount of protein was separated using 10% SDS–PAGE and then transferred onto PVDF membranes, blocked with 5% nonfat milk for nonspecific binding, and incubated with primary antibodies at 4 °C overnight. Subsequently, the blots were incubated with secondary antibodies for 1 h at room temperature. Finally, immune complexes were visualized using the Bio-Rad ChemiDoc XRS system. GAPDH was used to normalize the expression of the proteins. The experiment was conducted in triplicate. The following primary antibodies were used: anti-granzyme B (1:10,000, Abcam, ab134993), anti-perforin (1:500, Santa Cruz, sc-373,943), anti-INHBC (1:2000, Sangon, D120862-0100), anti-CD63 (1:2000, Sangon, D198650), anti-CD9 (1-800, Abcam, ab223052), and anti-GAPDH (1:50,000, Proteint, 60004-1-Lg). The following secondary antibodies were used: goat anti-mouse IgG H&L (HRP) (1:10,000, Abcam, ab205719) and goat anti-rabbit IgG H&L (HRP) (1:10,000, Abcam, ab6721).

### Labeling of exosomes and tracing the NK92-MI uptake of labeled exosomes

1,1′-Dioctadecyl-3,3,3′,3′-tetramethylindocarbocyanine perchlorate (Dil; Beyotime, Shanghai, China) was used to indicate the endocytosis of exosomes by NK92-MI cells through staining of the cytoplasm and intracellular membrane. Briefly, the SW480-exosome suspension was incubated with Dil (1:2000, Sigma) and washed through Exosome Spin Columns (MW3000, Life, Thermo Fisher, USA) to obtain Dil-labeled SW480 exosomes, which were incubated with NK92-MI cells. The chamber cells were fixed with 4% paraformaldehyde and stained with 4′,6-diamidino-2-phenylindole (DAPI; Beyotime). Finally, the endocytosis of the recipient cells was examined under a fluorescence microscope (Nikon ECLIPSE C1) equipped with a DS-U3 imaging system (Nikon).

### CCK-8 assay

The effect of CRC-derived exosomes on NK92-MI cell viability was determined using the Cell Counting Kit-8 (CCK-8) assay (Beyotime, C0038). The exosomes (50 µg/mL) isolated from SW480 cells were incubated with NK92-MI cells in 96-well plates for 48 h, followed by the addition of 10 µL of CCK-8 solution and further incubation for 0, 24, 48, 72, and 96 h. The absorbance was measured at 450 nm using a microplate reader.

### Cytotoxicity analysis of NK92-MI cells

Lactate dehydrogenase (LDH) release was used to determine the cytotoxicity of NK92-MI cells by assessing the integrity of the plasma membrane using the LDH kit (Beyotime) according to the manufacturer’s instructions. In brief, NK cells were pretreated with and without exosomes and then seeded together with tumor cells in culture media without PS and FBS. After co-culture for 24 h, the culture was centrifuged, and 120 µL of medium was collected and transferred to a new 96-well plate. The cells were lysed using the LDH release reagent, and the absorbance was measured at 490 nm using Epoch 2 (BioTek Instruments, USA).

### Enzyme-linked immunosorbent assay (ELISA)

NK92-MI cells were seeded in a 96-well plate at a density of 1 × 10^5^ cells/well. To explore the effect of exosomes on NK cells, in addition to NK92-MI cells, exosomes (50 µg/mL) were added to the 96-well plate. After co-culture for 24 h, the culture medium of NK92-MI cells was collected, and the supernatant was collected following centrifugation for 5 min at 1200×*g*. IFN-γ production by NK cells was detected using a Human IFN-r ELISA kit (Prod#EH6242M, Biotechwell, China) according to the manufacturer’s instructions.

### Immunofluorescence and immunohistochemistry (IHC)

CRC tissues and NK92-MI cells were fixed in 4% formalin at 4 °C for 8 h, embedded in paraffin blocks, and cut into 3-µm sections, which were mounted onto slides. The slides were then permeabilized in 0.2–0.5% Triton X-100 and blocked in 5% normal donkey serum at room temperature for 1 h. The slides were incubated with anti-perforin (1:250, Santa Cruz, sc-373,943) and anti-granzyme B (1:250, Abcam, ab134933) antibodies overnight and then incubated with DAPI and fluorescence-conjugated goat anti-mouse IgG H&L (1:500, Abcam, ab150117). Finally, the slides were fixed with fluorescence mounting medium (Sangon) and imaged using a Zeiss LSM880 NLO microscope. The CRC tissues were probed with an anti-NK1.1 monoclonal antibody (Thermo Fisher #16-5941) and examined using an Axiophot light microscope (Zeiss, Oberkochen, Germany).

### Whole transcriptome RNA sequencing of exosomes and bioinformatics analysis

Total RNA was isolated from exosomes derived from SW480 cells that had been induced (termed the EMT-exo group) or not induced by TGF-β (termed the non-EMT-exo group) using an Exosomal RNA Isolation Kit (NGB-58,000, Amyjet Scientific, China) according to the product specification. Then, ribosomal RNA was eliminated from RNA using the Ribo-zero Gold rRNA Removal Kit (Illumina, USA). First-strand cDNA was synthesized using random hexamer primers and SuperScript II and second-strand cDNA was synthesized using DNA Polymerase I and RNase H to construct cDNA libraries using the TruSeq Stranded RNA Sample Preparation Kit (Illumina, USA). The cDNA libraries were assessed using an Agilent Bioanalyzer 2100 system. High-throughput lncRNA sequencing was performed on a Hiseq ^TM^ 2500 platform (Illumina, USA) with a paired-end 150-bp read run at Yingbio Technology. Fast-QC (http://www.bioinformatics.babraham.ac.uk/projects/fastqc/) software was used for the quality control of raw reads. The gene expression was normalized by fragments per kilobase of exon per million fragments mapped. Using the DEGSeq algorithm to screen differentially expressed lncRNAs (DElncRNAs), the significance threshold was set at Log2FC > 1 or < − 1, and the false discovery rate was < 0.05. The target genes were predicted based on the DElncRNAs. The functional annotation of predicted DElncRNA targets was performed using Gene Ontology (GO) and the Kyoto Encyclopedia of Genes and Genomes (KEGG) analysis. The sequencing coverage and quality statistics of the samples are summarized in Additional file 4: Table S2.

### Expression and prognosis analysis from The Cancer Genome Atlas (TCGA) database

The transcriptome sequencing count files of 473 colon adenocarcinoma (COAD) tissues and 41 adjacent tissues were downloaded from the Genomic Data Commons (GDC) website (https://portal.gdc.cancer.gov). These data were used to analyze the differential expression of lncRNA SNHG10 by EdgeR. The clinical data of the COAD samples (n = 453) was obtained from TCGA website (https://www.cancer.gov/tcga), and data pertaining to the M stage with metastasis (n = 111) were selected for survival analysis.

### Lentivirus vector construction and transfection

Next, 0.75 µg of the target plasmid pLVX-CMV-lncRNA-EGFP-IRES-Puro (Addgene, China), 0.75 µg of psPAX2 (Addgene), and 0.5 µg of pMD2.G (Addgene) were mixed and co-transfected into 293FT cells (which were used to package lentiviruses) using the Effectene Transfection Reagent (QIAGEN, Germany; 301,425). The supernatant of 293FT cells was collected and filtered to harvest the lentivirus. Next, 30 µL of polybrene (10 mg/mL) and 150 µL of lentivirus were added to a 6-well plate containing SW480 cells. Finally, stable cell lines were obtained by selection with puromycin and examined using cell immunofluorescence.

The knockdown of inhibin subunit beta C (*INHBC*) expression was performed using a siRNA purchased from GenePharma (China). INHBC siRNA or control siRNA (5 µL of siRNA was diluted in 45 µL of OPTI-MEM (CORNING, China)) was transfected into SW480 cells using Lipofectamine™ 2000 (Invitrogen). The sequences of the siRNAs are listed in Additional file 3: Table S1.

### Transcriptome sequencing and bioinformatics analysis

Transcriptome sequencing was performed in triplicate in NK92-MI cells after incubation with exosomes (50 µg/mL) loaded with blank vector (termed Vector exo 1, 2, and 3) or overexpressed lncRNA vector (termed oe-lnc-SHNG10 exo 1, 2, and 3) for 24 h. The Illumina TruSeq mRNA Library Prep Kit was used to construct RNA sequencing libraries according to the manufacturer’s instructions. Yingbio Technology was then commissioned to perform RNA sequencing on an Illumina HiSeq 2500 platform with a paired-end 150-bp read run. The results of the quality control of raw reads, differentially expressed gene (DEG) analysis, and GO and KEGG analyses were consistent with the RNA sequencing described above. The sequencing coverage and quality statistics for each sample are summarized in Additional file 5: Table S3.

### Xenograft mice

All mouse experiments were approved and monitored by The Ethics Committee of Guangzhou First People’s Hospital. Healthy male BALB/c mice were purchased from JunKeBiological Co., Ltd., housed in the same environment, allowed to eat and drink water freely, and randomly divided into two groups (five mice per group). In vivo interference of tumor formation by exosomes was performed according to the method of Wang et al. [21]. After the mice were reared adaptively for 1 week, 5 × 10^6^ SW480 cells were suspended in 200 µL of precooled PBS and subcutaneously injected into the mice on the right side of the back. Tumor cells were transplanted and exosome application was started on the same day. Exosomes were injected into mice through the tail vein, twice a week, at a dose of 6 µg/injection. One group was injected with exosomes derived from LV-lncRNA-SW480 cells, whereas the other group was injected with exosomes derived from LV-vector-SW480 cells. Tail vein injection of exosomes was continued for 4 consecutive weeks. The growth rate of tumors was determined by measuring the tumor size at a specified time point. On the 8th day, the tumor length, width, and height were measured, and the tumor volume was calculated; these parameters were then measured every 3 days. Tumor volume (mm^3^) was calculated as (length × width^2^)/2. After 4 weeks, the mice were euthanized, and their plasma and tumor tissues were collected. For euthanization, the mice were anesthetized via inhalation of 2% isoflurane, followed by inhalation of a high concentration of isoflurane (5%); the mice lost consciousness rapidly and were decapitated.

### Flow cytometry

NK cells in xenograft mice pre-conditioned with exosomes were evaluated using flow cytometry. Briefly, cells were collected and suspended in 200 µL of PBS, followed by incubation with NK1.1 monoclonal antibody (16-5941, Thermo Fisher Scientific) at room temperature in the dark for 20 min. Subsequently, the cells were resuspended in 200 µL of PBS and examined on a BD FACSVerse™ apparatus (Becton Dickinson, USA) for flow cytometry sorting.

### Statistical analysis

Statistical analysis was performed on SPSS version 16.0 using one-way analysis of variance (ANOVA) following Tukey’s test to assess the significant differences between three or more groups. A *t *test was used for analyses between two groups. All data were presented as the mean ± standard deviation (SD). Significance was set at *p* < 0.05.

## Results

### Characteristics of EMT-induced exosomes from CRC cells

**Fig. 1:**
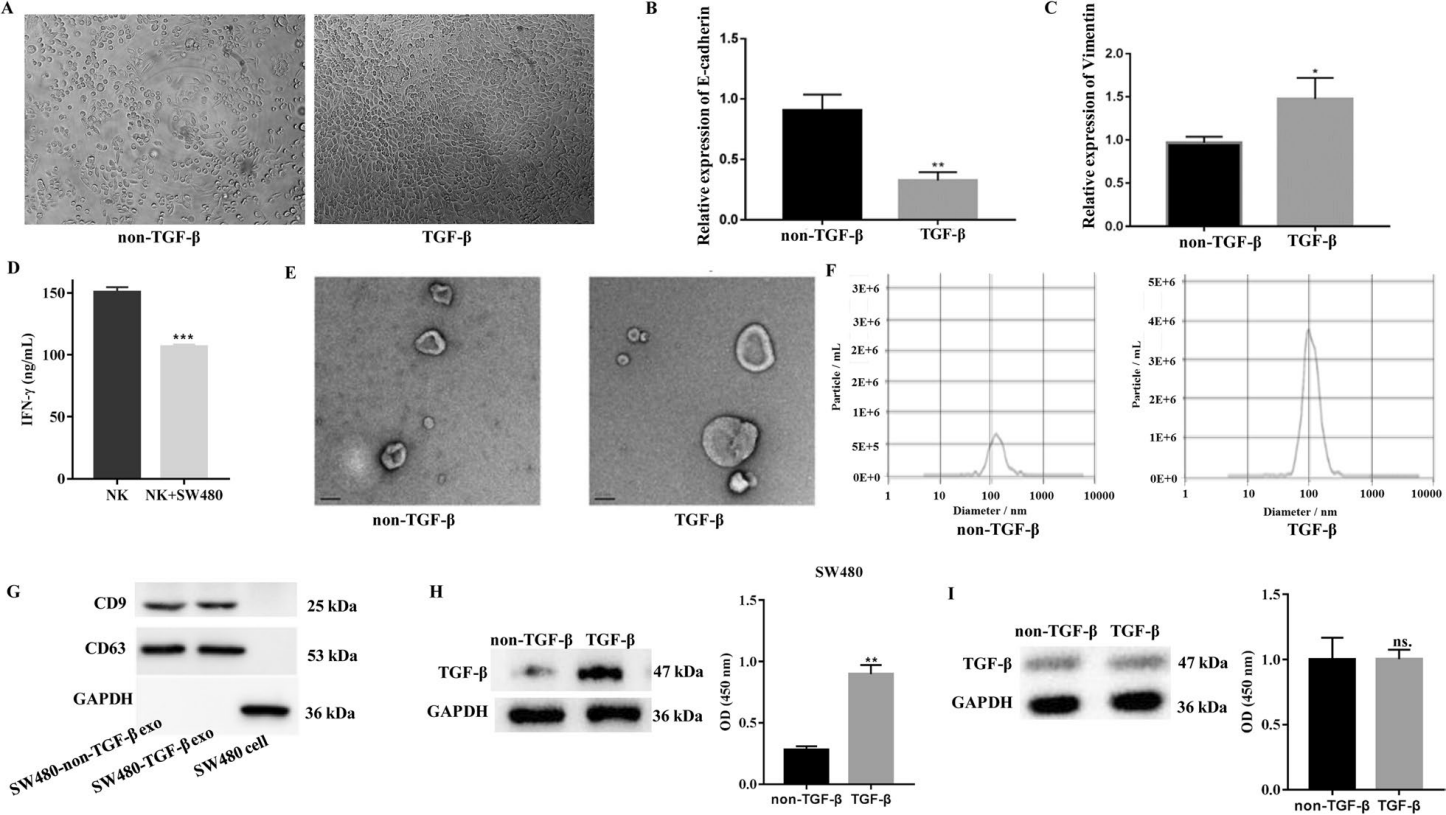
EMT-induced exosome characteristics of CRC cells.  **A** Representative image of the morphology of SW480 cells after TGF-β-induced EMT. The expression levels of E-cadherin (**B**) and VIM (**C**) in SW480 cells treated with TGF-β were detected by qRT-PCR. **D** Production of IFN-γ from NK92-MI cells when cultured alone or co-cultured with SW480 cell. **E** Transmission electron microscopy of exosomes derived from blank or TGF-β-induced SW480 cells. Scale bar, 100 nm. **F** The size and distribution of particles were detected by nanoparticle-tracking analysis. **G** The expression of the CD63 and CD9 biomarker in exosomes derived from SW480 cells was determined by western blotting. **H** Expression of TGF-β in SW480 cells induced by TGF-β or not. **I** Expression of TGF-β in exosomes derived from SW480 cells induced with TGF-β or not. *GAPDH* was used to normalize gene expression. *t*-test, *P < 0.05, **P < 0.01

To obtain metastatic CRC cells, we cultured SW480 cells in the presence of TGF-β to construct an EMT model. Compared with the non-TGF-β control, SW480 cells displayed a clear change from an epithelial to a mesenchymal morphology after treatment with TGF-β (Fig. 1A). A significant decrease in the expression of E-cadherin (an epithelial marker) and an increase in the expression of vimentin (a mesenchymal marker) were detected using qRT-PCR following treatment with TGF-β (Fig. 1B, C). These results are consistent with the findings of previous studies [22, 23] and suggest that the EMT model of SW480 CRC cells induced by TGF-β was successfully established. A previous study showed that cancer cells can regulate NK cell function, such as educating NK cells to a metastasis-promoting cell state [8]. Furthermore, IFN-γ production from NK cells was downregulated by the culture medium of SW480 cells (Fig. 1D), suggesting that these cells suppress NK cell function. We focused on examining the manner in which SW480 cells depressed NK cells. Exosomes are the most suspected entities because they usually act as functional transmitters between cells. Thus, exosomes were isolated from TGF-β-induced or non-induced SW480 cells. Particle morphology was evaluated using TEM, as shown in Fig. 1E, and particles with a mean diameter of 100 ± 53.8 nm were observed only after treatment with TGF-β, as determined by NTA (Fig. 1F). The western blotting results suggested that both induced and non-induced particles positively expressed CD63 and CD9, which are exosomal membrane markers (Fig. 1G). Collectively, these results indicated that particles derived from TGF-β-treated SW480 cells exhibited typical exosome characteristics.

To eliminate the interference of the exogenous application of TGF-β, we examined the levels of TGF-β in SW480 cells and exosomes in the presence or absence of TGF-β, respectively. The levels of TGF-β in SW480 cells were strongly upregulated in the TGF-β application group, whereas the TGF-β levels between the two groups of exosomes were not different compared with that in the non-TGF-β group (Fig. 1H, I). This indicates that the exogenous application of TGF-β does not promote the encapsulation event by exosomes; thus, the effect of TGF-β was ruled out.

### EMT-exo derived from CRC cells inhibited the function of NK cells

**Fig. 2:**
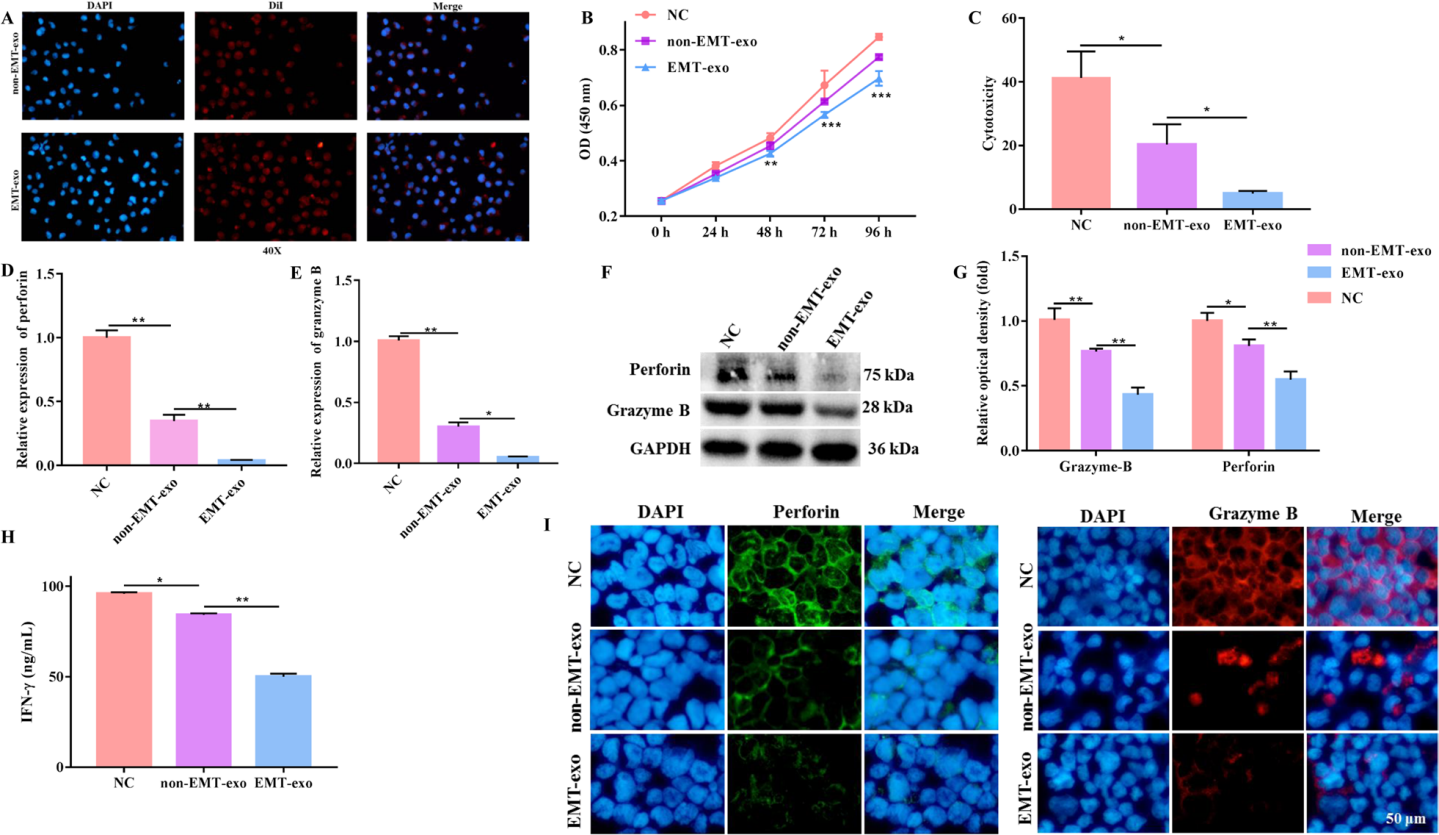
EMT-exo inhibited the function of NK92-MI cells. **A** Representative fluorescence microscopy image of the Dil-labeled exosomes (red) internalized by NK92-MI cells. **B** The viability of NK92-MI cells was detected by the CCK-8 assay. **C** The cytotoxicity of NK92-MI cells (pretreated with EMT-exo or not) co-cultured with SW480 cells was detected by the LDH assay. The expression of the toxic molecules perforin and granzyme B in NK92-MI cells (pretreated with EMT-exo or not) co-cultured with SW480 cells was measured by qRT-PCR (**D**, **E**) and western blotting (**F**, **G**). **H** The production of IFN-γ from NK92-MI cells (pretreated with EMT-exo or not) co-cultured with SW480 cells was detected by ELISA. **I** The expression of the toxic molecules perforin and granzyme B in NK92-MI cells was measured by immunofluorescence. *GAPDH* was used to normalize gene expression. The data were analyzed by ANOVA followed by Tukey’s test, *P < 0.05, **P < 0.01

We defined exosomes secreted by EMT SW480 cells induced by TGF-β as EMT-exo and those not induced by TGF-β as non-EMT-exo. We then investigated whether EMT-exo affected NK cell function. As shown in Fig. 2A, EMT-exo and non-EMT-exo labeled with Dil were internalized by NK92-MI cells, as observed using fluorescence microscopy. Furthermore, to investigate the effect of EMT-exo on the proliferation of NK92-MI cells, we co-cultured NK92-MI cells with EMT-exo and non-EMT-exo. The CCK-8 assay results revealed that EMT-exo significantly decreased the proliferation of NK92-MI cells (Fig. 2B) compared with that of cells without exosome incubation (NC group) or non-EMT-exo treatment.

Then, we assessed the manner in which exosomes affected the cytotoxicity of NK cells. We pretreated NK cells with non-EMT-exo, EMT-exo, or PBS and then seeded them together with SW480 cells in the medium. The LDH assay also demonstrated that exosomes significantly impaired the cytotoxicity of NK92-MI cells compared with the NC group and that the cytotoxicity was significantly lower in EMT-exo-treated NK92-MI cells than in NK cells pretreated with non-EMT-exo (Fig. 2C). Consistent with the LDH assay results, the release of the major toxic molecules perforin and granzyme B and IFN-γ production from NK92-MI cells were significantly decreased following exosome treatment, as measured using ELISA, qRT-PCR, and western blotting (Fig. 2D, H). Moreover, the release of perforin and granzyme B and IFN-γ production were significantly downregulated following EMT-exo treatment compared with that following non-EMT-exo treatment (Fig. 2D, H). The immunofluorescence experiment yielded the same results: the fluorescence intensity was reduced in the non-EMT-exo-treated group compared with that in the control group, and a more significant decrease was observed after EMT-exo treatment (Fig. 2I). These results collectively suggest that EMT-exo impairs the NK cell function.

### Identification of candidate functional lncRNAs in EMT-exo

**Fig. 3:**
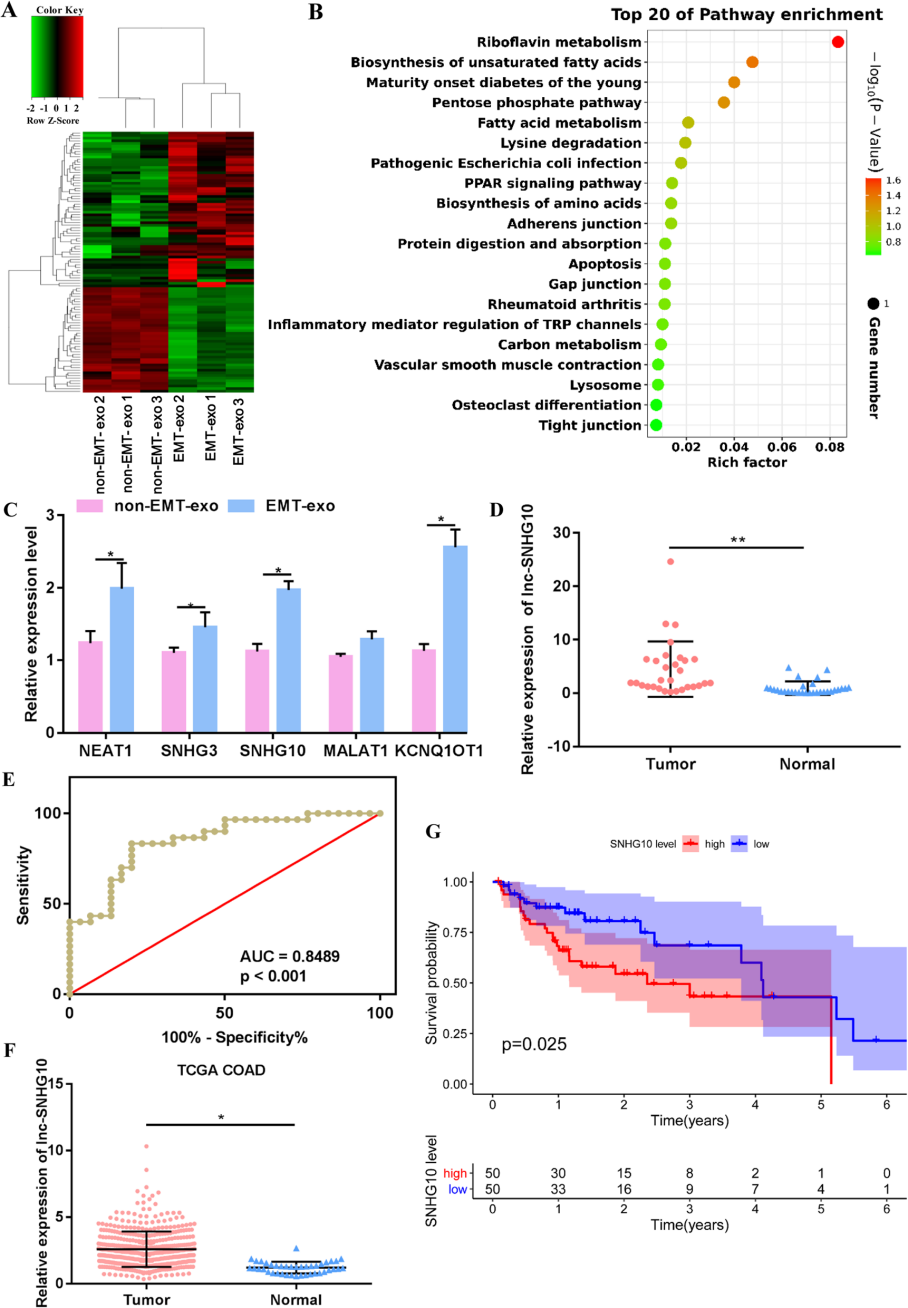
EMT exosomal lncRNA profile. **A** Heatmap diagram of differentially expressed lncRNAs (DElncRNAs) between EMT-exo and non-EMT-exo. Red, upregulated; green, downregulated. **B** Top 20 KEGG enriched pathways. Pathways are indicated on the left, enrichment is indicated on the right, and the size of the circle is proportional to the number of genes. **C** Validation of five selected DElncRNAs. NEAT1, nuclear paraspeckle assembly transcript 1; SNHG3, small nucleolar RNA host gene 3; SNHG10, small nucleolar RNA host gene 10; MALAT1, metastasis-associated lung adenocarcinoma transcript 1; KCNQ1OT1, KCNQ1 opposite strand/antisense transcript 1. **D** Expression of lncRNA SNHG10 in CRC tumor tissues (n = 30) and adjacent normal tissues (n = 30). **E** Receiver operating characteristic (ROC) curve of lncRNA SNHG10. The area under the ROC curve was 0.8489 (95% confidence interval 0.7524–0.9454). **F** Expression level of lncRNA SNHG10 in colon adenocarcinoma tissues (COAD, n = 473) and adjacent tissues (n = 41) from the GDC database. **G** Analysis of the prognosis of lncRNA SNHG10 in COAD (n = 453) from the TCGA database. *t*-test, *P < 0.05, **P < 0.01

Previous study demonstrated that lncRNAs can be loaded by exosomes to realize functional transfer [24]. Thus, we hypothesized that EMT-exo impairs the function of NK cells by transporting lncRNAs from CRC cells to NK cells. In this study, EMT-exo and non-EMT-exo were sequenced to screen for DElncRNAs as potential target functional molecules. A total of 95 DElncRNAs were obtained, including 39 downregulated and 56 upregulated DElncRNAs identified in EMT-exo compared with that in control exosomes (Fig. 3A). All these DElncRNAs were mainly involved in riboflavin metabolism, the PPAR signaling pathway, and the tight junction pathway and were enriched in the GO term of negative regulation of EMT (Fig. 3B, Additional file 1: Fig. S1). The five significantly upregulated DElncRNAs were used for qRT-PCR validation (small nucleolar RNA host gene 3 [SHNG3], SHNG10, nuclear paraspeckle assembly transcript 1 [NEAT1], metastasis associated lung adenocarcinoma transcript 1 [MALAT1], and KCNQ1 opposite strand/antisense transcript 1 [KCNQ1OT1]). As shown in Fig. 3C, with the exception of SHNG3, the expressions of the other four lncRNAs were significantly different, including that of SNHG10, whose expression was significantly upregulated in the EMT-exo group compared with that in the non-EMT-exo group. SNHG10 exhibited the lowest *P* value and the highest abundance in the RNA sequencing data. Lan et al. had demonstrated that SNHG10 promotes hepatocarcinogenesis and metastasis [25]. Therefore, SNHG10 was initially used as a candidate lncRNA. To further assess the prognostic value of SNHG10, we assessed its expression in 30 CRC tumor tissues and 30 paired paracancerous tissues. Its expression was significantly upregulated in cancerous tissues compared with that in adjacent tissues (Fig. 3D). A receiver operating characteristic curve of SNHG10 showed that the area under the curve was 0.8489 (95% confidence interval, 0.7524–0.9454) and had a significant predictive value for distinguishing CRC tumor tissues from adjacent tissues (Fig. 3E). Moreover, data from TCGA database supported our results that SNHG10 was highly expressed in tumors and was associated with poor prognosis (Fig. 3F, G). Collectively, these findings reveal that SNHG10 is highly expressed in EMT-exo and that SNHG10 expression is higher in CRC tumors than in normal tissues; therefore, the expression levels of SNHG10 could be used as a prognostic marker.

### Exosomes inhibited NK cell function through SNHG10

**Fig. 4:**
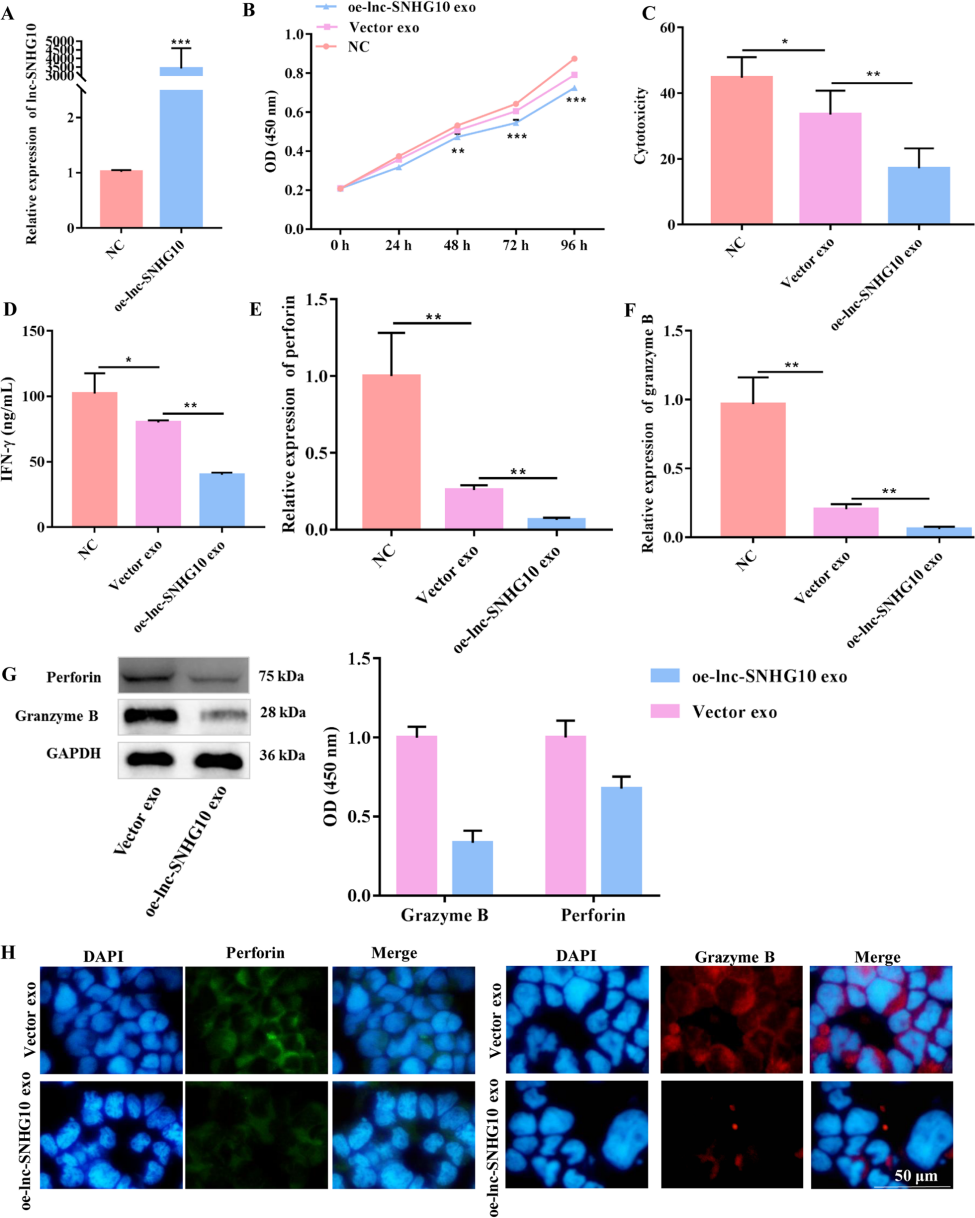
EMT exosomal lncRNA SNHG10 inhibited NK cell cytotoxicity. **A** The efficacy of the overexpression of the lncRNA SNHG10 in SW480 cells was verified by qRT-PCR. **B** The viability of NK92-MI cells was detected by CCK-8 assay. **C** The cytotoxicity of NK92-MI cells (pretreated with EMT-exo or not) co-cultured with SW480 cells was detected by LDH assay. **D** The production of IFN-γ from NK92-MI cells was detected by ELISA. The expression of the toxic molecules perforin and granzyme B in NK92-MI cells (pretreated with EMT-exo or not) co-cultured with SW480 cells was measured by qRT-PCR (**E**, **F**), western blotting (**H**), and immunofluorescence (**H**). *GAPDH* was used to normalize gene expression. *t*-test, *P < 0.05, **P < 0.01

To further verify whether the suppressive effects of EMT-exo on NK92-MI cells were mediated in an SNHG10-dependent manner, SNHG10-overexpressing SW480 cells were established using a lentiviral vector. The overexpression efficiency of SNHG10 was confirmed using qRT-PCR (Fig. 4A) and fluorescence imaging (Additional file 2: Fig. S2). Exosomes derived from EMT SW480 cells overexpressing SNHG10 (oe-lnc-SNHG10 exo) were co-cultured with NK92-MI cells. Compared with NK92-MI cells cultured alone, NK92-MI cells co-cultured with vector exo (defined as exosomes derived from EMT SW480 cells transfected with a blank lentiviral vector) had significantly suppressed viability and cytotoxicity (Fig. 4B, C). Importantly, the EMT exosomal SNHG10 overexpression led to more significant impairment of viability and cytotoxicity than that in the Vector exo group (Fig. 4B, C). IFN-γ production in NK92-MI cells was also largely suppressed by exosomal SNHG10 overexpression (Fig. 4D). Moreover, forced expression of EMT exosomal SNHG10 effectively suppressed the release of perforin and granzyme B, both at the mRNA and protein levels, in NK92-MI cells compared with that in the Vector exo group (Fig. 4E, G). The fluorescence intensity of perforin and granzyme B in NK92-MI cells was also stronger in the Vector exo group than in the SNHG10-overexpressing group (Fig. 4H). These results suggest that lncRNA SNHG10 in EMT-exo impaired NK cell function.

### EMT exosomal SNHG10 targeted INHBC and activated the TGF-β signaling pathway in NK cells

**Fig. 5:**
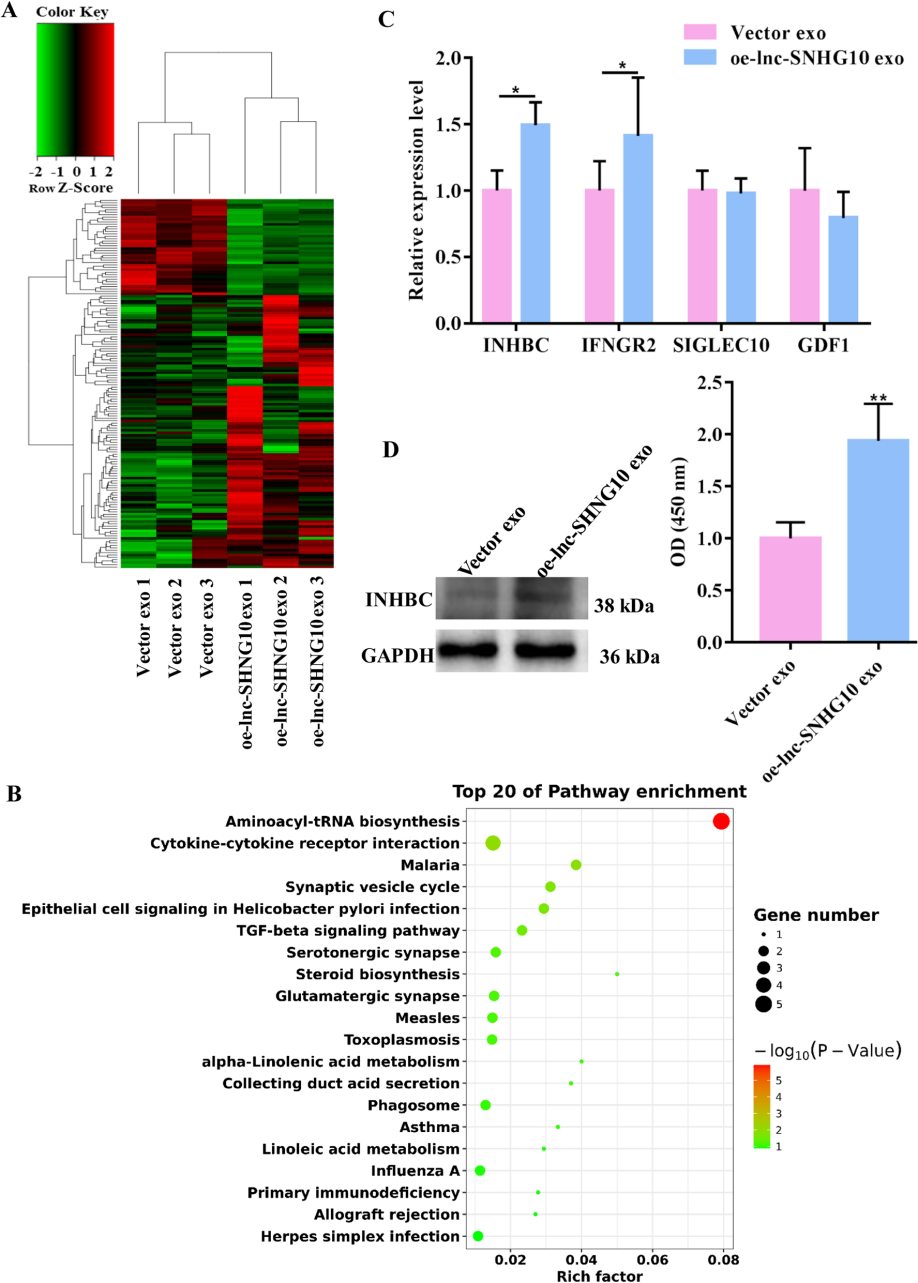
LncRNA SNHG10-mediated regulation of INHBC. **A** Heatmap diagram of DEGs in NK92-MI cells between two groups: one group was incubated with exosomes derived from SW480 cells transfected with vector (Vector exo), whereas the other group was incubated with exosomes derived from SW480 cells that overexpressed lncRNA SNHG10 (oe-lnc-SNHG10 exo). Red, upregulated; green, downregulated. **B** Top 15 upregulated KEGG enrichment pathways. The KEGG pathways are indicated on the left, the *P* values are indicated on the right. **C** qRT-PCR validation of four selected DEGs between the Vector exo group and oe-lnc-SNHG10 exo group. **D** Protein level of INHBC in NK92-MI cells in the Vector exo group or oe-lnc-SNHG10 exo group. *GAPDH* was used to normalize gene expression. *t*-test, *P < 0.05, **P < 0.01

To further investigate the mechanism underlying the EMT exosomal SNHG10-mediated inhibition of NK cells, transcriptome sequencing was performed for NK92-MI cells co-cultured with vector exo and oe-lnc-SNHG10 exo. Incubation with oe-lnc-SNHG10 exo generated 154 DEGs, of which 40 were downregulated and 114 were upregulated in NK cells compared with those in the Vector exo group (Fig. 5A). Considering that high SNHG10 expression is related to the immunosuppression of NK cells, we focused on the pathways involved in the regulation of NK cytotoxicity. As shown in Fig. 5B, oe-lnc-SNHG10 exo significantly activated aminoacyl-tRNA biosynthesis and the TGF-β signaling pathway. Among these, the TGF-β signaling pathway has been shown to inhibit NK cell metabolism and function by affecting glycolytic capacity, oxidative phosphorylation, and respiratory capacity [26]. Therefore, in this study, the scope of potential target genes was initially restricted to those involved in the TGF-β signaling pathway.

We selected four DEGs that had been reported to promote cancer progression and showed positive response to SNHG10 abduction for verification. The qRT-PCR results showed that compared with that in the Vector exo group, INHBC and interferon gamma receptor 2 (IFNGR2) were significantly upregulated in the oe-lnc-SNHG10 exo group, whereas sialic-acid-binding Ig-like lectin 10 (SIGLEC10) and growth differentiation factor 1 (GDF1) showed no statistically significant difference between two groups (Fig. 5C). Among these, INHBC showed the highest significant difference and was involved in the TGF-β signaling pathway. Thus, we speculated that INHBC is a receptor molecule for SNHG10-regulated NK cells. Western blotting results showed that oe-lnc-SNHG10 exo significantly upregulated INHBC expression in NK92-MI cells, which confirmed our hypothesis (Fig. 5D). Therefore, SNHG10 upregulated the expression of INHBC, which is involved in the TGF-β signaling pathway in NK cells.

### Exosomal lncRNA SNHG10 regulated the function of NK cells through INHBC

**Fig. 6:**
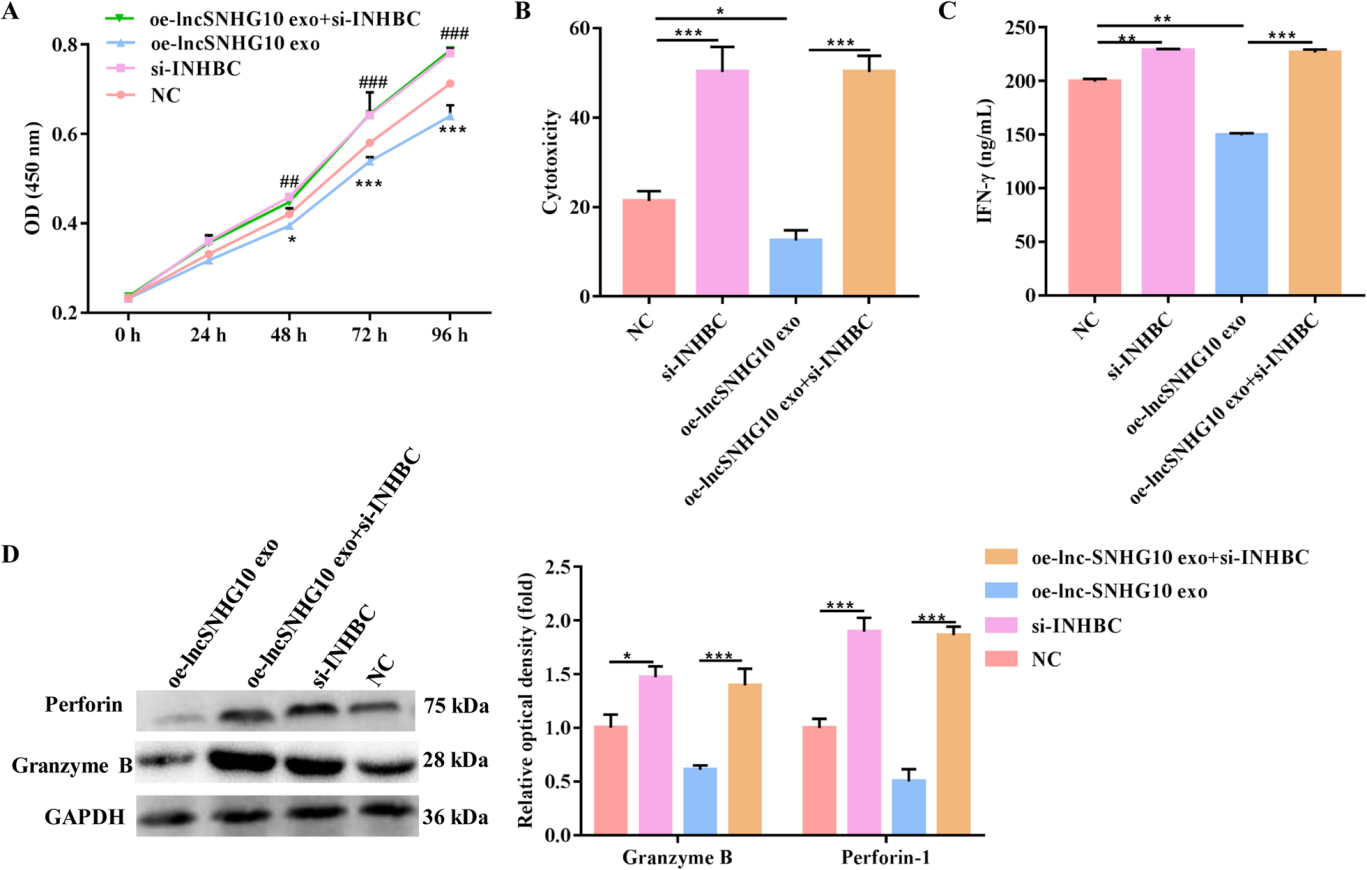
Exosomal lncRNA SNHG10 regulated the function of NK cells through INHBC. **A** The proliferation of NK92-MI cells was detected by CCK-8 assay. **B** The cytotoxicity of NK92-MI cells was detected by LDH assay. **C** The production of IFN-γ from NK92-MI cells was detected by ELISA. **D** The expression of the toxic molecules perforin and granzyme B in NK92-MI cells was measured by western blotting. *GAPDH* was used to normalize gene expression. The data were analyzed by ANOVA followed by Tukey’s test, *NC vs. oe-lncSNHG10 exo, # NC vs. si-INHBC. *P < 0.05, **P < 0.01, ***P < 0.001, ##*P* < 0.01, ###*P* < 0.001

INHBC, which is a member of the TGF-β superfamily, is homologous to TGF-β [27]. To determine whether SNHG10 regulates NK cell function by upregulating INHBC expression, we performed INHBC loss-of-function studies in the context of SNHG10 overexpression. The results revealed that oe-lnc-SNHG10 exo led to a significant decrease in cell proliferation and cytotoxicity and that this stimulatory effect was largely eliminated when si-INHBC was co-transfected into NK92-MI cells (Fig. 6A, B). Similarly, IFN-γ production and the release of perforin-1 and granzyme B from NK92-MI cells were significantly upregulated by si-INHBC, whereas they were downregulated by oe-lnc-SNHG10 exo; however, co-treatment of oe-lnc-SNHG10 exo and si-INHBC largely rescued these alterations in NK92-MI cells when induced alone (Fig. 6C, D). Therefore, INHBC suppressed NK cell function and si-INHBC treatment abrogated the effect of SNHG10 overexpression on NK cells.

### Exosomal SNHG10 promoted CRC growth by inhibiting NK cell growth in vivo

**Fig. 7:**
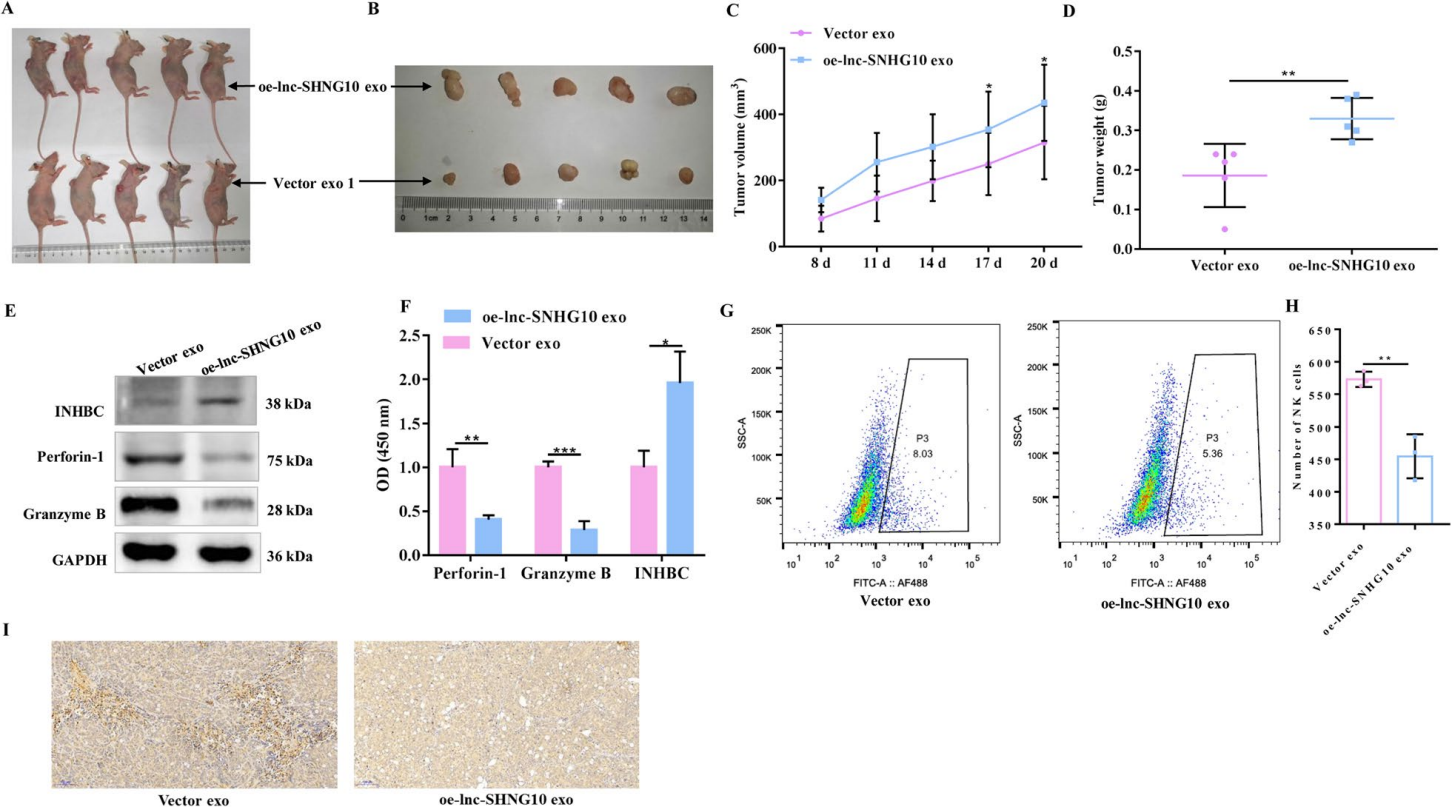
Exosomal lncRNA SNHG10 promoted CRC growth by inhibiting NK cells in vivo. **A**, **B** Xenograft tumors of BALB/C mice after injection of LV-lncRNA SNHG10-SW480 and LV-vector-SW480 cells (n = 10 per group). **C** Tumor volume in the two groups. **D** Tumor weight of each mouse in the two groups. **E**, **F** The levels of the INHBC, perforin, and granzyme B proteins in mice were detected by western blotting. Representative images (**G**) and statistical analysis (**H**) of the flow cytometry of NK1.1 in mice in the two groups.**I** Representative images of IHC of NK1.1 in mice in the two groups. *GAPDH* was used to normalize gene expression. *t*-test, *P < 0.05, **P < 0.01

To investigate the tumor growth-promoting potential of exosomal SNHG10 in vivo, we constructed the LV-lncRNA SNHG10-SW480 and LV-vector-SW480 cell lines with stable SNHG10 overexpression and then collected exosomes. These exosomes, which carried LV-lncRNA and LV-vector, were intravenously injected into BALB/C mice, and tumor tissues were collected after 4 weeks (Fig. 7A, B). As indicated by the tumor volume and weight, tumors in the oe-lnc-SNHG10 exo group were significantly larger than those in the LV-vector exosome group (Fig. 7C, D). These results suggest that exosomal SNHG10 overexpression promotes tumor growth. In addition, LV-lncRNA SNHG10 exosomes significantly increased the expression of INHBC and suppressed the release of perforin-1 and granzyme B in mice (Fig. 7E, F). The positive rate of NK1.1-labeled NK cells in mice decreased in the oe-lnc-SNHG10 exo group, as confirmed using flow cytometry (Fig. 7G, H) and IHC (Fig. 7I). These results demonstrate that exosomal SNHG10 promotes tumor growth by inhibiting NK cells in vivo.

## Discussion

The engineering of evasion of immune surveillance is an essential requirement for tumor survival and metastasis [28]. In recent years, increasing evidence has demonstrated that lncRNAs are involved in immune regulation by tumor cells, as reported by Zhou et al., who showed that lincRNA-p21 in breast cancer cells could reverse the function of macrophages to facilitate the development of breast cancer [29]. The highly expressed lncRNA HOTAIR led to a decrease in T-lymphocyte proliferation activity and NK cell activity during leukemia [30]. The long-distance transport of lncRNAs usually depends on extracellular vesicles. The biological molecular mechanism underlying the immunosuppression of NK cells by tumor cells with EMT at a distance is not completely understood. Here, we constructed an EMT model of CRC cells and found that EMT-derived exosomes carried SNHG10 and upregulated INHBC expression, resulting in the repression of NK cell function.

Exosomes from EMT-induced CRC cells significantly inhibited the viability of NK cells as well as perforin-1 and granzyme B secretion. Consistent with our results, a previous study reported that exosome-mediated intercellular communication was involved in the modulation of NK cell response, although this communication may not be the main role of exosomes, but is driven by the cargo they carry [19]. Hepatocellular carcinoma cell-derived exosomal circUHRF1 was shown to induce NK cell exhaustion and decrease NK cell tumor infiltration [31]. Li et al. found that exosomal linc-EPHA6-1 could regulate the cytotoxicity of NK cells by sponging hsa-miR-4485-5p [32]. These results explain our observation that the number of NK cells in tumor infiltrates was reduced and that NK cell function was suppressed after treatment with EMT exosomes. Moreover, we found that SNHG10 was highly expressed in CRC cells and overexpression of EMT exosomal SNHG10 significantly suppressed NK cell function, thereby contributing to tumor cell growth *in vitro* and in vivo. Although there are relatively few functional research reports on SNHG10, their results are consistent with our results. Lan et al. evidenced that SNHG10 can promote hepatocarcinogenesis and metastasis through a positive feedback loop [25]. Jiang found that SNHG10, SNHG12, and LINC00115 were abnormally expressed in bladder cancer and that downregulation of SNHG12 expression was related to the inhibition of tumor proliferation [33]. In summary, these evidences support our results and suggest that EMT-exo promotes the growth of CRC cells by inhibiting NK cell function via the transport of SNHG10 both *in vitro* and in vivo.

Transcriptome sequencing revealed that INHBC is a potential target for SNHG10, and qRT-PCR results showed that compared with healthy controls, INHBC was highly expressed in patients with CRC and was associated with poor prognosis. INHBC is an inhibin that belongs to a branch of the TGF-β superfamily, and a previous study reported that INHBC may be involved in the pathogenesis and malignant transformation of the human endometrium [34]. INHBC may also induce the secretion of IL-6 and TGF-β and promote the proliferation and inhibit the apoptosis of renal cells during diabetic nephropathy [27]. Therefore, INHBC is believed to be associated with the alteration of the malignant phenotype of cells, which is consistent with the present findings.

Moreover, the expression of INHBC, which is involved in the TGF-β signaling pathway [35], was significantly induced by SNHG10. INHBC is homologous to TGF-β, which has been recognized as an immune suppressor by involving the development, differentiation, tolerance induction, and homeostasis of immune cells to regulate the progression of human diseases [36]. Fujii et al. showed that TGF-β1 induces the downregulation of activation markers and cytotoxic granules in NK cells, including CD226, NKG2D, NKp30, perforin, and granzyme B, through Smad2/3 signaling [37]. In colon cancer models, the administration of LY2157299, an inhibitor of the TGF-β receptor kinase, could mitigate the cytotoxicity of NK cells and inhibit liver metastases [38]. As a homologous counterpart of TGF-β, we inferred that INHBC may have functions similar to those of TGF-β. Consequently, these findings supported the conclusion that lncRNA SNHG10 ultimately leads to NK cell dysfunction probably via INHBC.

## Conclusions

EMT CRC cell-derived exosomes inhibit NK cell function and promote tumor growth by transporting lncRNA SNHG10. Mechanistically, lncRNA SNHG10 upregulated the expression of INHBC to activate the TGF-β signaling pathway, thus leading to the suppression of NK cytotoxicity. Our study provides evidence of the crosstalk between CRC and NK cells, possibly laying the foundation for new immunotherapeutic strategies for patients with CRC.

## Supplementary Information

**Additional file 1: Fig. S1.** Top 20 GO enrichment of DElncRNAs. The left indicates GO terms, the right indicates enrichment, and the size of the solid circle indicates the number of genes. 

**Additional file 2: Fig. S2.** Overexpression of lncRNA SNHG10 in SW480 cells observed by fluorescence.

**Additional file 3: Table S1.** Information of the primers used in this study.

**Additional file 4: Table S2.** Quality control of clean data and statistics of RNA sequencing.

**Additional file 5: Table S3.** Quality control of clean data and statistics of transcriptome sequencing.

## Data Availability

The RNA sequencing data generated in this study are available in the Sequence Read Archive (SRA) under accession number PRJNA679092 on the NCBI. Other data that support the findings of this study are included within the article. Please contact the author for data requests.

## Abbreviations

COAD: Colon adenocarcinoma
CRC: Colorectal cancer
DAPI: 4′,6-Diamidino-2-phenylindole
DEG: Differentially expressed gene
Dil: 1,1′-Dioctadecyl-3,3,3′,3′-tetramethylindocarbocyanine perchlorate
EMT: Epithelial–mesenchymal transition
EMT-exo: EMT-derived exosomes
INHBC: Inhibin subunit beta C
LDH: Lactate dehydrogenase.
lncRNA: Long noncoding RNA
NTA: Nanoparticle-tracking analysis
NK: Natural killer
PBS: Phosphate-buffered solution
TCGA: The Cancer Genome Atlas
TEM: Transmission electron microscope
TGF-β: Transforming growth factor beta
GO: Gene Ontology
KEGG: Kyoto Encyclopedia of Genes and Genomes
SNHG: Small nucleolar RNA host gene
MCP-1: Monocyte chemoattractant protein 1
STR: Short tandem repeat
ELISA: Enzyme-linked immunosorbent assay
IHC: Immunohistochemistry
GDC: Genomic Data Commons
TCGA: The Cancer Genome Atlas
SD: Standard deviation
NEAT1: Nuclear paraspeckle assembly transcript 1
MALAT1: Metastasis associated lung adenocarcinoma transcript 1
KCNQ1OT1: KCNQ1 opposite strand/antisense transcript 1
IFNGR2: Interferon gamma receptor 2
SIGLEC10: Sialic-acid-binding Ig-like lectin 10
GDF1: Growth differentiation factor 1
SRA: Sequence Read Archive
