# Correction to: Exosomal lncRNA SNHG10 derived from colorectal cancer cells suppresses natural killer cell cytotoxicity by upregulating INHBC

**DOI:** 10.1186/s12935-022-02470-9

**Published:** 2022-02-02

**Authors:** Yiwen Huang, Yanbo Luo, Wentao Ou, Yuanyuan Wang, Dong Dong, Xiaowen Peng, Yuqi Luo

**Affiliations:** 1 Department of Emergency, Nansha Hospital, Guangzhou First People’s Hospital, School of Medicine, Southern China University of Technology, Guangzhou, Guangdong China; 2 Department of Gastrointestinal and Hepatobiliary Surgery, Shenzhen Longhua District Central Hospital, Longhua District, No. 187, Mission Avenue, Shenzhen, 518110 Guangdong China; 3 Department of General Surgery, Nansha Hospital, Guangzhou First People’s Hospital, School of Medicine, Southern China University of Technology, Guangzhou, Guangdong China; 4 Department of Neurology, Nansha Hospital, Guangzhou First People’s Hospital, School of Medicine, Southern China University of Technology, Guangzhou, Guangdong China; 5 Department of Laboratory Medicine, Nansha Hospital, Guangzhou First People’s Hospital, School of Medicine, Southern China University of Technology, Guangzhou, Guangdong China

## Correction to: Cancer Cell Int (2021) 21:528 https://doi.org/10.1186/s12935-021-02221-2

In the article [1], the authors have found an error of Granzyme B and GAPDH in Fig. 4G. This error was caused by the same group name and we put a wrong picture in Fig. 4G during figure processing, but the original picture that we first submitted to the journal was correct. The correct Fig. 4G is given in this correction:

**Fig. 4:**
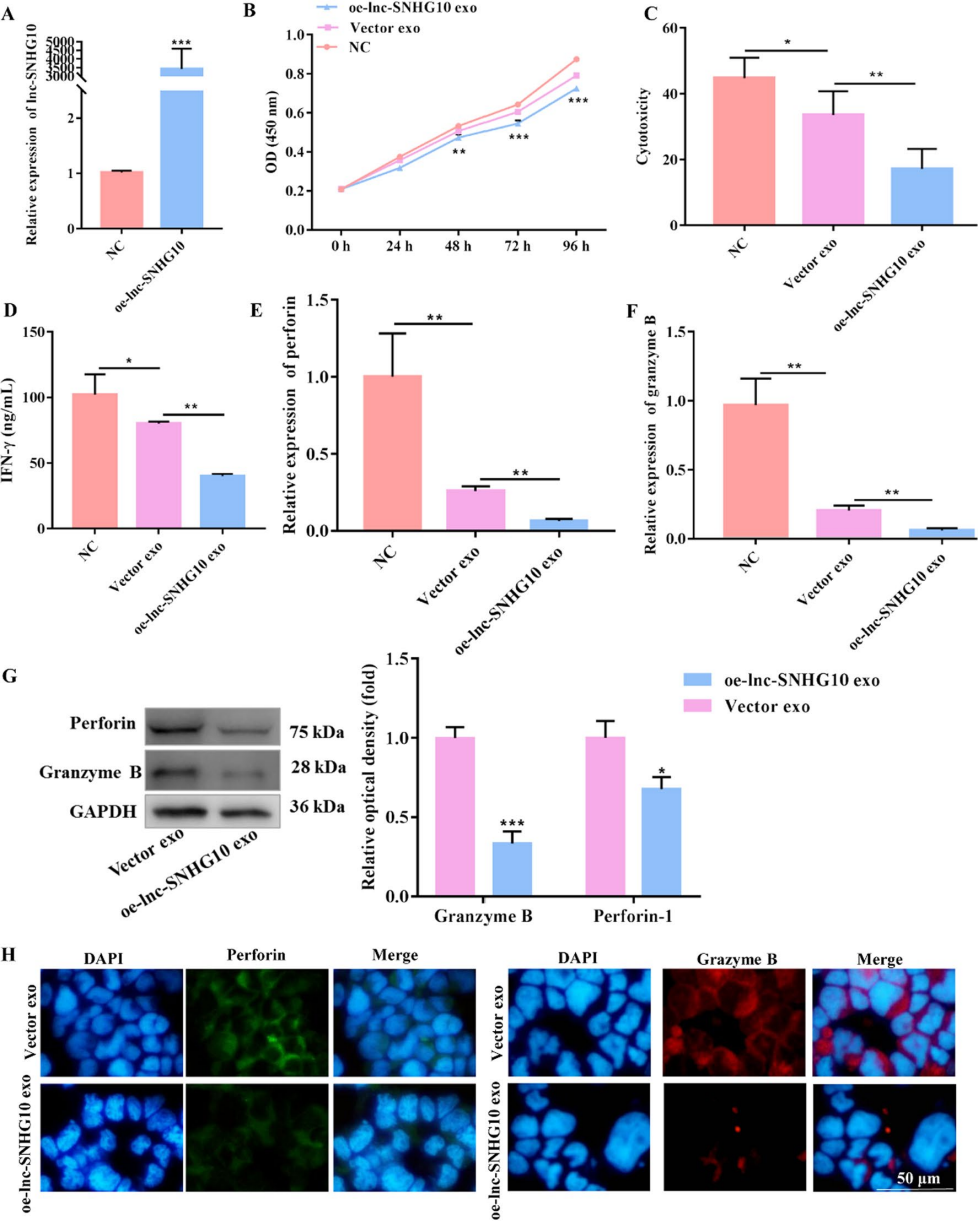
EMT exosomal lncRNA SNHG10 inhibited NK cell cytotoxicity.** A** The efficacy of the overexpression of the lncRNA SNHG10 in SW480 cells was verified by qRT-PCR.** B** The viability of NK92-MI cells was detected by CCK-8 assay.** C** The cytotoxicity of NK92-MI cells (pretreated with EMT-exo or not) co-cultured with SW480 cells was detected by LDH assay.** D** The production of IFN-γ from NK92-MI cells was detected by ELISA. The expression of the toxic molecules perforin and granzyme B in NK92-MI cells (pretreated with EMT-exo or not) co-cultured with SW480 cells was measured by qRT-PCR (**E**,** F**), western blotting (**G**), and immunofluorescence (**H**). GAPDH was used to normalize gene expression. t-test, *P < 0.05, **P < 0.01, ***P < 0.001
