# Supplementary figures and images for: Exosomal lncRNA SNHG10 derived from colorectal cancer cells suppresses natural killer cell cytotoxicity by upregulating INHBC
Source: Cancer Cell Int. 2021 Oct 12;21:528. doi: 10.1186/s12935-021-02221-2 (PMC8507338; doi:10.1186/s12935-021-02221-2)

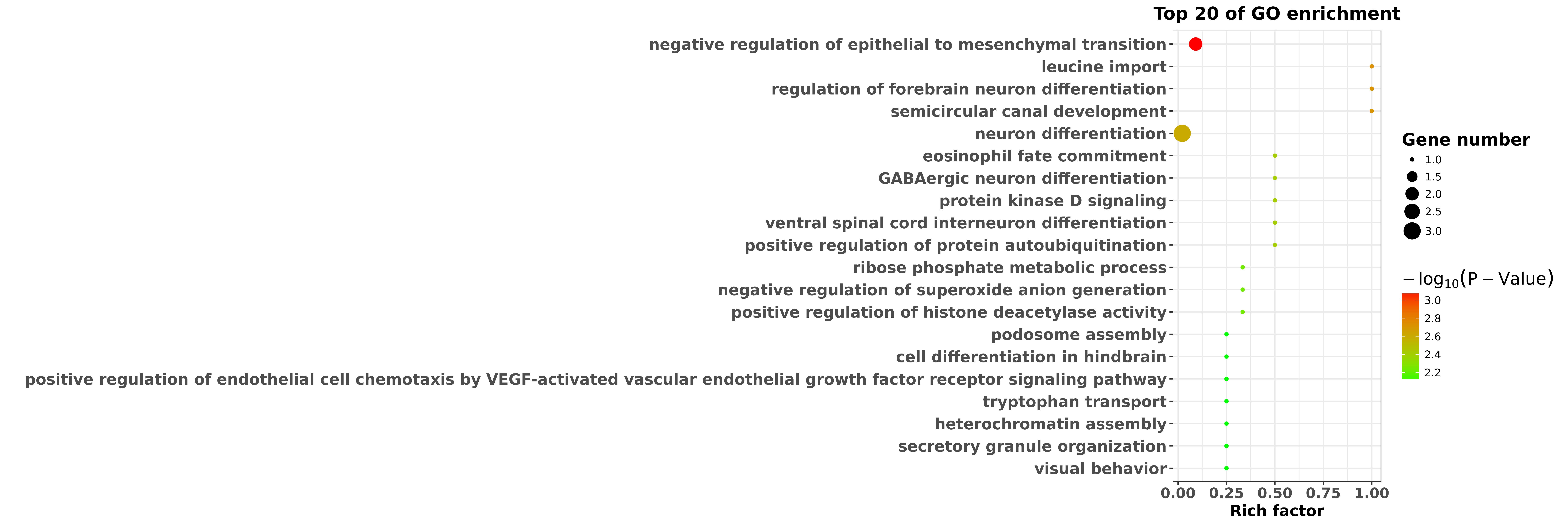

Supplement: Supplementary file 1 — Additional file 1: Fig. S1. Top 20 GO enrichment of DElncRNAs. The left indicates GO terms, the right indicates enrichment, and the size of the solid circle indicates the number of genes. [file 12935_2021_2221_MOESM1_ESM.tif]

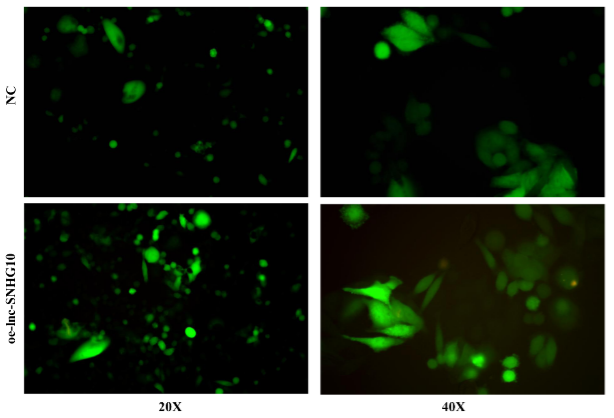

Supplement: Supplementary file 2 — Additional file 2: Fig. S2. Overexpression of lncRNA SNHG10 in SW480 cells observed by fluorescence. [file 12935_2021_2221_MOESM2_ESM.tif]
