# Supplementary material for: Exosomal lncRNA SNHG10 derived from colorectal cancer cells suppresses natural killer cell cytotoxicity by upregulating INHBC
Source: Cancer Cell Int. 2021 Oct 12;21:528. doi: 10.1186/s12935-021-02221-2 (PMC8507338; doi:10.1186/s12935-021-02221-2)
Supplement: Supplementary file 3 — Additional file 3: Table S1. Information of the primers used in this study. [file 12935_2021_2221_MOESM3_ESM.docx]

Table S1. The sequences of primers and siRNAs used in the study.

| Name | Accession number of the targeted mRNA | Sequences (5’ – 3’) | Size (bp) |
| --- | --- | --- | --- |
| GAPDH-F | NM_001357943.2 | AGAAGGCTGGGGCTCATT | 158 |
| GAPDH-R |  | TGCTAAGCAGTTGGTGGTG |  |
| Perforin-F | NM_005041.6 | CGCTTCTACAGTTTCCATGTGGT | 131 |
| Perforin-R |  | CCGTAGTTGGAGATAAGCCTGAG |  |
| Granzyme B-F | NM_001346011.2 | TCTCCAACGACATCATGCTACTG | 168 |
| Granzyme B-R |  | GTAGTGTGTGTGAGTGTTTTCCCA |  |
| INHBC-F | NM_005538.4 | GCCTCTCCACCATCAACCAG | 96 |
| INHBC-R |  | AGAACATGAGACTGGCCTGC |  |
| IFNGR2-F | NM_001329128.2 | GTCCAGGCACAACTGCTTTG | 94 |
| IFNGR2-R |  | TGGAGGCATCTGCCATTGTT |  |
| SIGLEC10-F | NM_001322105.2 | CTTCTTTCTCCTGGACGGGG | 70 |
| SIGLEC10-R |  | ACTGAGAAGTGGGAGGTCGT |  |
| GDF1-F | NM_001387440.1 | GGGAGTATGACACAGCCGAG | 90 |
| GDF1-R |  | AGAAGCGCTTGTCCTTCACC |  |
| NEAT1-F | NR_131012.1 | AAACGCTGGGAGGGTACAAG | 71 |
| NEAT1-R |  | ATGCCCAAACTAGACCTGCC |  |
| lnc-SNHG3-F | NR_036473.1 | ATGGTAGCAACGGGAGGTTG | 107 |
| lnc-SNHG3-R |  | TCACGTCATGAGGCAAGCAA |  |
| lnc-MALAT1-F | NR_144568.1 | AGTTTGGTCTTGGGGTTTGGA | 93 |
| lnc-MALAT1-R |  | CTGTGTTATGCCTGGTTAGGTATGA |  |
| lnc-KCNQ1OT1-F | NR_002728.3 | GGAGTTTGCCTGAGTTGGGA | 78 |
| lnc-KCNQ1OT1-R |  | ACCTAAAACCACCATGCGGA |  |
| Vimentin-F | NM_003380.5 | AGTCCGCACATTCGAGCAAA | 114 |
| Vimentin-R |  | AACTTACAGCTGGGCCATCG |  |
| E-cadherin-F | NM_001387440.1 | CTGGCGTCTGTAGGAAGGCA | 155 |
| E-cadherin-R |  | GGGCAGTAAGGGCTCTTTGAC |  |
| lnc-SNHG10-F | NR_003138.3 | GCAAAACAAGAGGGAAGACGA | 74 |
| lnc-SNHG10-R |  | TCCCAGTTGTCTCGGCGTA |  |
| INHBC siRNA | NM_005538.4 | GCAACAUUGUCAAGACUGACAUCAGUCUUGACAAUGUUGCUG | 42 |
| siRNA NC |  | UUCUCCGAACGUGUCACGUTTACGUGACACGUUCGGAGAATT | 42 |
