# Supplementary material for: Exosomal lncRNA SNHG10 derived from colorectal cancer cells suppresses natural killer cell cytotoxicity by upregulating INHBC
Source: Cancer Cell Int. 2021 Oct 12;21:528. doi: 10.1186/s12935-021-02221-2 (PMC8507338; doi:10.1186/s12935-021-02221-2)
Supplement: Supplementary file 4 — Additional file 4: Table S2. Quality control of clean data and statistics of RNA sequencing. [file 12935_2021_2221_MOESM4_ESM.docx]

Table S2. Quality control of clean data and statistics of RNA sequencing

| Samples | Total Reads Filter | Reads Filter (%) | Total Bases Filter | Bases Filter (%) | GC (%) | Mapped Rate (%) |
| --- | --- | --- | --- | --- | --- | --- |
| non-EMT-exo 1 | 92979446 | 0.840 | 13909881694 | 0.838 | 63 | 0.718 |
| non-EMT-exo 2 | 72400854 | 0.849 | 10831424194 | 0.847 | 63 | 0.733 |
| non-EMT-exo 3 | 91475208 | 0.836 | 13689531870 | 0.834 | 62 | 0.750 |
| EMT-exo 1 | 64301004 | 0.867 | 9619192976 | 0.865 | 59 | 0.558 |
| EMT-exo 2 | 87954740 | 0.860 | 13151409579 | 0.857 | 57 | 0.489 |
| EMT-exo 3 | 83771058 | 0.871 | 12534375315 | 0.869 | 59 | 0.581 |

Group of EMT-exo means that exosomes derived from SW480 cells that had been induced by TGF-β.

Group of non-EMT-exo means that exosomes derived from SW480 cells that had not been induced by TGF-β.
