# Supplementary material for: Exosomal lncRNA SNHG10 derived from colorectal cancer cells suppresses natural killer cell cytotoxicity by upregulating INHBC
Source: Cancer Cell Int. 2021 Oct 12;21:528. doi: 10.1186/s12935-021-02221-2 (PMC8507338; doi:10.1186/s12935-021-02221-2)
Supplement: Supplementary file 5 — Additional file 5: Table S3. Quality control of clean data and statistics of transcriptome sequencing. [file 12935_2021_2221_MOESM5_ESM.docx]

Table S3. Quality control of clean data and statistics of transcriptome sequencing

| Samples | Total Reads Filter | Reads Filter (%) | Total Bases Filter | Bases Filter (%) | GC (%) | Mapped Rate (%) |
| --- | --- | --- | --- | --- | --- | --- |
| Vector exo 1 | 60571142 | 0.931 | 9074583553 | 0.930 | 50 | 0.948 |
| Vector exo 2 | 62981770 | 0.906 | 9433985051 | 0.905 | 51 | 0.946 |
| Vector exo 3 | 76086886 | 0.918 | 11398211931 | 0.916 | 50.5 | 0.954 |
| oe-lnc-SHNG10 exo 1 | 56074422 | 0.924 | 8400197801 | 0.923 | 50 | 0.943 |
| oe-lnc-SHNG10 exo 2 | 76171720 | 0.925 | 11411045308 | 0.924 | 50.5 | 0.944 |
| oe-lnc-SHNG10 exo 3 | 62743110 | 0.927 | 9399642456 | 0.926 | 50 | 0.945 |

Group of Vector exo means that NK92-MI cells co-incubated with exosomes loaded with blank vector.

Group of oe-lnc-SHNG10 exo means that NK92-MI cells co-incubated with exosomes loaded with overexpressed lncRNA vector.
